# Correction: A biparatopic HER2-targeting ADC constructed *via* site-specific glycan conjugation exhibits superior stability, safety, and efficacy

**DOI:** 10.1039/d6cb90040b

**Published:** 2026-10-09

**Authors:** Qi Xue, Jianjian Peng, Wenying Dai, Qingsong Wu, Jinbiao Jiao, Yudi Hu, Wanxing Sha, Yang Yang, Wenhao Yu, Siyang Liu, Ting Xu, Jie P. Li

**Affiliations:** a State Key Laboratory of Coordination Chemistry, School of Chemistry and Chemical Engineering, Chemistry and Biomedicine Innovation Center (ChemBIC), Nanjing University Nanjing China jieli@nju.edu.cn; b Jiangsu Alphamab Biopharmaceuticals Co., Ltd., BioBay, Building C23 218 Xinghu Street, Suzhou Industrial Park Suzhou Jiangsu China tingxu@alphamabonc.com

## Abstract

Correction for ‘A biparatopic HER2-targeting ADC constructed *via* site-specific glycan conjugation exhibits superior stability, safety, and efficacy’ by Qi Xue *et al.*, *RSC Chem. Biol.*, 2025, **6**, 1284–1296, https://doi.org/10.1039/D5CB00096C.

The authors regret two instances of incorrectly depicted chemical structures.

One instance is featured in Fig. 1A and 2A, the graphical abstract and the supplementary information file under the heading ‘Chemical synthesis,’ and refers to the incorrect depiction of the stereochemical configuration of the DXd payload.

A second instance is in Fig. 2A where one methylene group (–CH_2_–) is omitted between the triazole ring and the amide carbonyl in the GalNAz-derived linkage.

The correct versions of Fig. 1 and 2 are shown here. The conclusions of the paper have not been affected. All compounds prepared and used in the study, the synthetic experiments, reaction conditions, analytical characterization and mass spectrometric results reported in the published article remain unchanged.

**Fig. 1:**
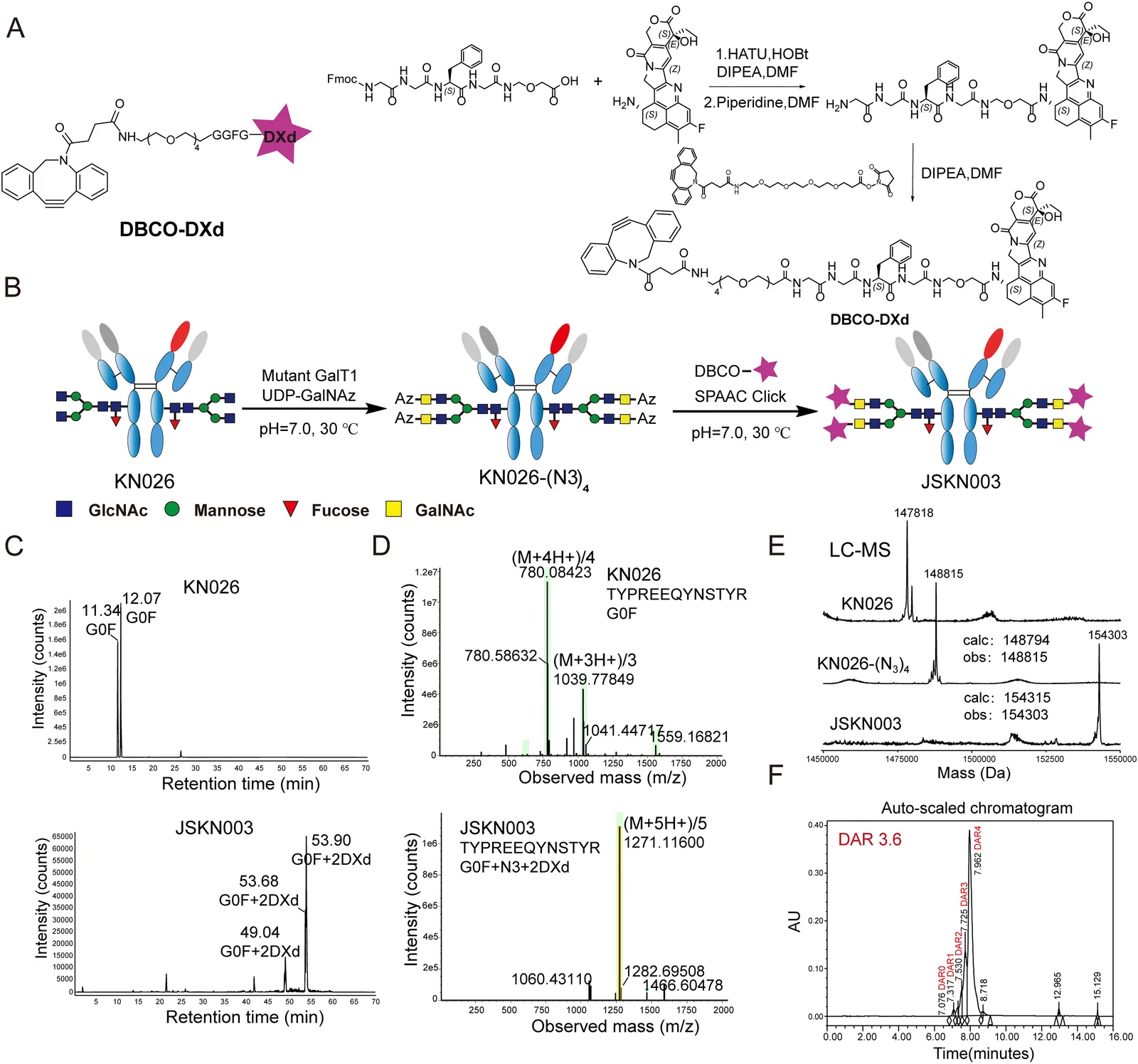
Development of JSKN003 *via* site-specific Fc glycosylation modification and molecular characterization. (A) Structure and synthesis route of DBCO-DXd. (B) Schematic representation of the site-specific chemoenzymatic synthesis of JSKN003 (illustrating the reaction scheme of glycans present in over 85% of the antibody population). (C) Extracted ion chromatograms (EIC) comparing the *N*-glycopeptide profiles of KN026 and JSKN003. (D) Electrospray ionization tandem mass spectrometry (ESI-MS/MS) fragmentation of representative *N*-glycopeptides from KN026 and JSKN003, highlighting structural differences in glycoforms. (E) Electrospray ionization mass spectrometry (ESI-MS) analysis of intact antibodies: KN026, KN026-(N_3_)_4_ and JSKN003. (F) Auto-scaled chromatographic analysis of JSKN003.

**Fig. 2:**
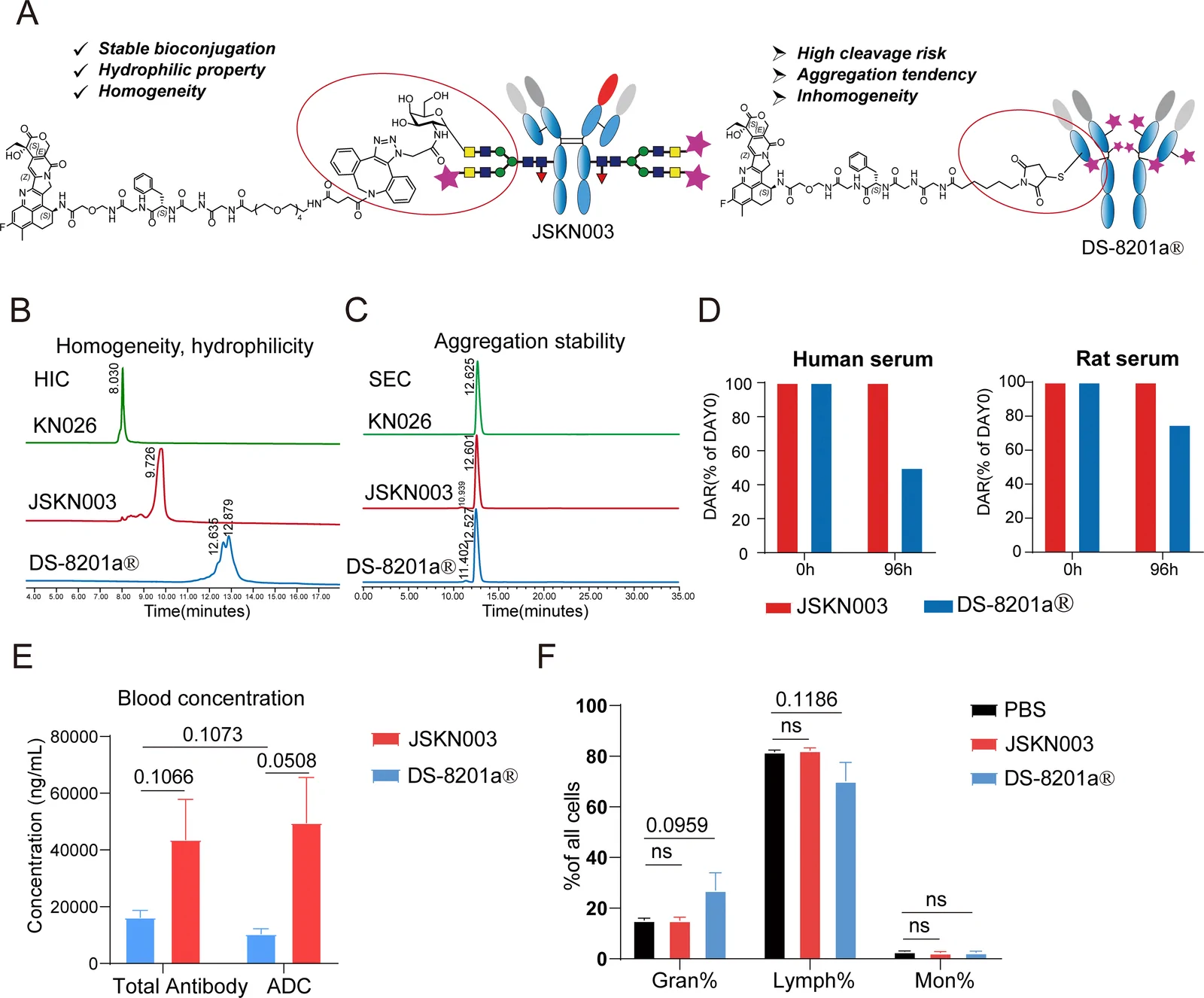
Biophysical and serum stability comparison between JSKN003 and DS-8201a. (A) Structural comparison between JSKN003 (glycoengineered ADC) and DS-8201a (commercial trastuzumab deruxtecan). (B) and (C) Hydrophobic interaction chromatography (HIC) (B) and size exclusion chromatography (SEC) (C) analysis comparing DS-8201a, KN026, and JSKN003. (D) Liquid chromatography-mass spectrometry (LC-MS) analysis demonstrating the stability of JSKN003 and DS-8201a in human and rat serum. Bar graphs show changes in drug-to-antibody ratio (DAR) values at 0 and 96 hours. Error bars are not shown as the measurements represent single technical replicates due to limited sample availability. (E) Pharmacokinetic analysis of DS-8201a and JSKN003 in tumor-bearing BALB/c mice. (F) Hematological toxicity assessment following a 20 mg kg^−1^ dose of ADC. Neutropenia, lymphadenopathy, and changes in monocyte levels were observed in mice. Three to four animals were administered JSKN003, DS-8201a, or PBS, and hematological markers were subsequently analyzed. Data are presented as mean ± SEM; *n* = 3. Student’s *t* test. n.s, not significant; *P < 0.05.

The Royal Society of Chemistry apologises for these errors and any consequent inconvenience to authors and readers.

